# Transcriptomics Advancement in the Complex Response of Plants to Viroid Infection

**DOI:** 10.3390/ijms23147677

**Published:** 2022-07-12

**Authors:** Melissa Joubert, Noëlani van den Berg, Jacques Theron, Velushka Swart

**Affiliations:** 1 Hans Merensky Chair in Avocado Research, Forestry and Agricultural Biotechnology Institute, University of Pretoria, Pretoria 0002, South Africa; melissa.joubert@fabi.up.ac.za (M.J.); noelani.vdberg@fabi.up.ac.za (N.v.d.B.); 2 Department of Biochemistry, Genetics and Microbiology, Faculty of Natural and Agricultural Sciences, University of Pretoria, Pretoria 0002, South Africa; jacques.theron@up.ac.za

**Keywords:** viroids, noncoding RNA, host response, transcriptome studies, RNA-seq, gene expression, viroid–host interactions

## Abstract

Viroids are the smallest plant pathogens, consisting of a single-stranded circular RNA of less than 500 ribonucleotides in length. Despite their noncoding nature, viroids elicit disease symptoms in many economically important plant hosts, and are, thus, a class of pathogens of great interest. How these viroids establish disease within host plants, however, is not yet fully understood. Recent transcriptomic studies have revealed that viroid infection influences the expression of genes in several pathways and processes in plants, including defence responses, phytohormone signalling, cell wall modification, photosynthesis, secondary metabolism, transport, gene expression and protein modification. There is much debate about whether affected pathways signify a plant response to viroid infection, or are associated with the appearance of disease symptoms in these interactions. In this review, we consolidate the findings of viroid–host transcriptome studies to provide an overview of trends observed in the data. When considered together, changes in the gene expression of different hosts upon viroid infection reveal commonalities and differences in diverse interactions. Here, we discuss whether trends in host gene expression can be correlated to plant defence or disease development during viroid infection, and highlight avenues for future research in this field.

## 1. Introduction

Viroids are small (246–434 nt), single-stranded RNAs which are responsible for diseases in many economically important crops. More than 30 species of viroids have been discovered to date, and these have been grouped into two viroid families—the *Pospiviroidae* and *Avsunviroidae*. These families are distinguished based on conserved sequence motifs present in viroid genomes, their mode of replication and the secondary structure they form through internal base-pairing [1]. Disease symptoms caused by viroids include, amongst others, stunting, leaf malformations, chlorosis of leaves, stems and/or fruit, the cracking of bark and shortening or thickening of stems [1]. Due to their noncoding nature, it is assumed that viroids induce disease symptoms by recruiting host factors in planta. It has been hypothesised that pathogenesis involves the direct interaction of viroid RNA with host proteins or RNA. Alternatively, host RNA silencing machinery may also produce viroid-derived small RNAs (vd-sRNAs), which can interfere with various gene expression pathways within the infected plant. This has recently been reviewed [2,3,4,5].

Advancements in the field of genomics and transcriptomics have created new avenues for research into the molecular mechanisms of viroid disease. Microarray analyses have been used to study global changes in host gene expression induced by the potato spindle tuber viroid (PSTVd) [6], citrus exocortis viroid (CEVd) [7] and peach latent mosaic viroid (PLMVd) [8]. Subsequently, the advent of RNA sequencing technologies has allowed researchers to identify differentially expressed genes in PSTVd-infected tomato using the first RNA sequencing-based analysis in viroid research [9]. This paved the way for numerous studies that used RNA sequencing to investigate host responses to the hop stunt viroid (HSVd) [10], chrysanthemum stunt viroid (CSVd) [11], CEVd [12], hop latent viroid (HLV) and citrus bark cracking viroid (CBCVd) [13,14]. The accessibility of RNA sequencing has resulted in the rapid expansion of our knowledge of viroid–host interactions, since the availability of next-generation sequencing (NGS) technology allows for quick, high-throughput analyses of host gene expression [15,16]. This technology has the advantage of providing a global picture of how plant hosts respond to viroid infection, without the limitations of microarrays, which only measure the expression of genes for which probes have been specifically designed.

To date, the results of viroid–host transcriptome studies have not been consolidated in a single integrative review. The most recent review of plant transcriptomic responses to viroid infection was by Owens et al. [17], who reported on a limited number of these studies available at the time. More recently, Mihaljević et al. [18] reviewed phytohormone changes observed in viroid-infected hosts, though only four phytohormone pathways were discussed by the authors. In this review, we consider all the transcriptome data available for viroid–host interactions and contextualise these in terms of important plant processes. We discuss commonalities and discrepancies observed across different interactions for the expression of distinctive pathways and processes in plants. Though many of these processes are inter-related, they are examined separately in this review to provide an overview of how each of these processes is affected during viroid infection. Finally, we discuss possible implications for observable trends in terms of viroid pathogenesis and plant defence.

## 2. Viroids Activate Plant Immune Responses

Plants have evolved a complex immune response to combat diseases caused by pathogens. These responses include two lines of defence—pathogen-associated molecular pattern (PAMP)-triggered immunity (PTI), a basal defence response, and effector-triggered immunity (ETI), a more robust immune response [19]. Both types of immunity are associated with the accumulation of certain defence proteins, the activation of mitogen-activated protein kinases (MAPKs), as well as the production of reactive oxygen species (ROS), though the magnitude of the response differs between PTI and ETI [20]. Despite the non-living nature of viroids, host defence responses have been shown to be triggered in the hosts they infect (Table 1).

### 2.1. Pathogenesis-Related Proteins

One common host response to infection by plant pathogens is the increased accumulation of pathogenesis-related (PR) proteins as part of systemic acquired resistance (SAR) in plants [21,22,23]. Transcriptome studies of viroid–host interactions have revealed that several viroids trigger the increased expression of *PR* genes in various hosts, including tomato, cucumber, hop, avocado, peach, orchid and Etrog citron [10,12,13,14,24,25,26,27,28,29]. This corroborates early reports that PR proteins accumulated to higher levels in viroid-infected plants [30,31,32]. The activation of *PR* genes (which have varying functions in plant defence) in these interactions provides evidence for the activation of PTI in response to viroid infection.

**Table 1:** Molecular pathways with significant changes in gene expression after viroid infection of different hosts, as revealed by global transcriptome studies. Pathways with altered expression are indicated by + symbol, while pathways not mentioned to be altered in transcription are indicated by “NA”.

Viroid Species	Host Species	Tissue Type	Type of Transcriptome Analysis	Pathways Affected										Reference
				Plant Defense	Hormone Signalling	Secondary Metabolism	Cell Wall Modification	Photosynthesis	Transport	Transcription	RNA Silencing	Translation /Ribosome Biogenesis	Protein Modification/Degradation	
***Potato Spindle Tuber Viroid*** **(PSTVd)**	Tomato (*Solanum lycopersicum*)	Leaves	Microarray	+	+	NA	+	+	NA	NA	NA	+	+	[6]
		Leaves	RNA-Seq	+	+	NA	+	NA	+	NA	NA	NA	NA	[9]
		Leaves	Microarray	+	+	+	+	+	+	+	NA	+	+	[28]
		Roots	Microarray and RNA-Seq	+	+	+	+	+	+	+	NA	+	+	[24]
		Leaves	RNA-Seq	+	+	NA	+	+	+	+	+	+	+	[29]
	Potato (*Solanum tuberosum*)	Leaves and Tubers	Microarray	+	+	NA	+	+	+	+	NA	+	NA	[33]
		Whole plantlets	RNA-Seq	+	NA	+	NA	NA	+	NA	NA	NA	NA	[34]
	Pepper (*Capsicum annuum*)	Leaves	RNA-Seq	+	+	+	NA	+	+	+	NA	NA	NA	[35]
***Hop Stunt Viroid*** **(HSVd)**	Cucumber (*Cucumis sativus*)	Leaves	RNA-Seq	+	+	+	+	+	NA	+	+	+	+	[10]
	Sweet cherry (*Prunus avium*)	Fruit	RNA-Seq	+	+	+	NA	NA	+	+	NA	+	NA	[36]
**HSVd & *Hop Latent Viroid* (HLVd)**	Hop (*Humulus lupulus*)	Leaves	RNA-Seq	+	+	+	NA	+	+	+	NA	NA	+	[37]
***Peach Latent Mosaic Viroid*** **(PLMVd) ^1^**	Peach (*Prunus persica*)	Leaves	Microarray	+	+	NA	+	NA	NA	NA	NA	NA	NA	[8]
***Chrysanthemum Stunt Viroid*** **(CSVd)**	Chrysanthemum	Leaves	RNA-Seq	+	+	+	+	NA	+	+	NA	NA	+	[11]
***Citrus Exocortis Viroid*** **(CEVd)**	‘Etrog’ citron (*Citrus medica*)	Leaves	Microarray	+	NA	NA	+	+	+	NA	NA	NA	+	[7]
		Leaves	RNA-Seq	+	+	+	+	NA	NA	+	+	NA	+	[12]
***Citrus Bark Cracking Viroid*** **(CBCVd)**	Hop	Leaves	RNA-Seq	+	+	+	+	+	+	+	NA	+	+	[13]
**CBCVd and/or HLVd**	Hop	Leaves	RNA-Seq	+	+	+	+	+	+	+	NA	+	+	[14]
***Citrus Dwarfing Viroid*** **(CDVd)**	Sweet Orange (*Citrus sinensis*)	Stems	RNA-Seq	+	+	NA	NA	NA	+	+	NA	NA	+	[38]
	Trifoliate Orange (*Citrus trifoliata*)	Roots	RNA-Seq	+	+	+	NA	+	+	+	NA	NA	NA	[38]
***Dendrobium viroid*** **(DVd)**	Orchid (*Dendrobium officinale)*	Stems	RNA-Seq	+	+	+	NA	+	NA	+	NA	NA	NA	[25]

^1^ Study analysed the peach transcriptome altered in response to PLMVd, Peach Necrotic Ringspot Virus, or their dual infection. For the sake of consistency, only pathways affected by single PLMVd infection are noted here.

### 2.2. Resistance Proteins

A second plant defence response which is common to most plant–pathogen interactions is the production of resistance (R) proteins, which are usually expressed in plant hosts to detect pathogen effector proteins [19,39]. Several plant hosts upregulate the expression of *R* genes—such as the tomato mosaic virus resistance protein, RPM1 (resistance to *Pseudomonas syringae* pv. *maculicola* 1) and RPM-like proteins, DRLs (disease resistance-like proteins), RGAs (resistance gene analogues) and other nucleotide-binding site leucine-rich repeat proteins (NBS-LRRs)—upon viroid infection [9,12,13,24,25,29,33,34,36]. The upregulation of *R* genes in response to pathogens which are unable to express effector proteins is surprising, since it is doubtful that R proteins can directly recognise the viroid RNA. We would hypothesise that the increased expression of *R* genes in these interactions is an effect of the activation of linked defence responses (such as the upregulation of *WRKY*s), rather than a strategic counterattack by infected host plants.

### 2.3. Protein Kinases

Protein kinases regulate signalling pathways in all living organisms by altering the phosphorylation status of their target proteins in response to cellular signals [40,41]. One group of kinases that plays a crucial role in plant immunity is receptor-like kinases (RLKs), which bind to elicitors of pathogenic microorganisms. Following recognition, a signalling cascade is initiated to activate PTI pathways in the plant [42,43]. Viroid infection has been shown to increase the expression of the RLK, flagellin-sensing 2 (*FLS2*), in Etrog citron [12], cucumber [10] and tomato [24,29]. Góra-Sochacka et al. [24] also demonstrated that chitin elicitor receptor kinase 1 (*CERK1*) and EF-Tu receptor (*EFR*) genes were upregulated when tomato plants were infected with PSTVd.

The expression of pattern recognition receptors (*PRR*s) such as *FLS2* during viroid infection is an interesting observation, and may indicate that viroid RNA is somehow recognised as a PAMP by plant hosts—an idea which has been suggested by Flores et al. [3] and Navarro et al. [4]. This assertion was determined by those authors after it was revealed that double-stranded RNA (dsRNA) triggers PTI responses subsequent to its recognition by an unidentified receptor in Arabidopsis [44]. It must be noted that the upregulation of *PRR*s upon viroid infection is not necessarily correlated to an active function of RLKs during these interactions. Further studies are needed to investigate whether any of the upregulated receptors are able to recognise viroid dsRNA. The discovery of RLKs that bind to viroids in infected plants would provide a possible explanation for the activation of other PTI responses in these interactions.

As part of general PTI, members of the MAPK family are activated, and these kinases can then trigger other signalling cascades linked to defence responses [20,39]. A number of *MAPK* genes were upregulated in viroid-infected hop, Etrog citron, sweet cherry and tomato [6,9,10,12,13,24,28,29,36]. The cellular accumulation of these proteins is yet to be measured in viroid-infected hosts, but it is plausible that the increased activity of MAPKs may be responsible for setting off some of the immune responses observed in plant–viroid interactions.

### 2.4. Calcium Signalling

In addition to MAPK cascades, another cell signalling network important in plant immunity is one which uses calcium ions as a messenger molecule in both PTI and ETI responses [39,45]. Calcium signalling pathways use a collection of proteins common to plants, which include a set of calcium-dependent protein kinases (CDPKs), cyclic nucleotide-gated channels (CNGCs), calcium-binding calmodulin proteins (CaMs) and calmodulin-like proteins (CMLs) [45]. These proteins form networks which sense changing cellular calcium levels in stressed plants and, subsequently, activate additional host responses [46]. Most of the genome-wide transcriptomic studies explored in this review showed an upregulation of calcium signalling genes upon viroid infection [6,9,10,12,13,14,24,28,29,36]. To our knowledge, there have not yet been reports of the altered cellular calcium levels upon viroid infection. This should be investigated in future studies to confirm the inference of calcium signalling activation in these interactions. Since calcium networks can influence downstream immune responses, such as the transcriptional activation of defence genes, further evidence of modified calcium signalling would, to an extent, explain the upregulation of PTI and ETI pathway genes in viroid–host interactions.

### 2.5. Reactive Oxygen Species

Another downstream immune response influenced by calcium signalling is the production of ROS [46]. This group of compounds consists of several oxygen-containing molecules, which either act directly against invading plant pathogens or work by regulating various processes within plant cells [47,48]. While the accumulation of ROS as part of both PTI and ETI cannot be determined by transcriptomic studies, the expression of genes involved in ROS biosynthesis can be assessed. The most notable ROS biosynthesis proteins are NADPH oxidases, also known as respiratory burst oxidase homologs (RBOHs), and peroxidases [49]. Genes encoding both types of NADPH oxidases were upregulated in viroid–host interactions [10,12,14,28,29,33,36]. The transcriptional activation of ROS biosynthesis genes indicates that ROS might accumulate in infected hosts. However, this assertion would need to be confirmed by measurements of the amounts of ROS physically present in infected plant cells. Studies carried out in potato and *Solanum laxum*, for example, showed that, following infection with PSTVd, intracellular hydrogen peroxide levels increased and the activity of certain peroxidases was enhanced [50,51]. Additional investigations of ROS levels in viroid-infected hosts would reveal to what extent ROS accumulation forms a part of the host immune response in these interactions.

### 2.6. WRKY Transcription Factors

WRKY transcription factors (TFs) are a group of proteins which bind to promoter elements on the plant genome to modify the expression of host genes involved in various cellular processes, most notably those which play a role during plant–pathogen interactions [52,53]. Several WRKY TFs were upregulated after viroid infection of Etrog citron, cucumber, sweet cherry, tomato, hop, orchid and sweet pepper [9,10,12,14,24,25,28,35,36]. The altered expression of WRKY TFs in these interactions may, to some extent, explain the differential expression of various plant defence-related genes as discussed in this review. It is plausible that the upregulation of specific WRKY TFs at certain timepoints might be linked to the modified expression of their target genes. Should this be proven in future investigations, it would provide conclusive evidence to explain the mechanisms of immune pathway activation in viroid–host interactions.

### 2.7. Parallels in Host Immune Activation by Viroids

Taken together, transcriptome data from viroid-infected hosts reveal the upregulation of an abundance of plant defence genes across all interactions. This finding is rather curious when it is considered that viroids, unlike all other plant pathogens, do not encode proteins that could trigger PTI and/or ETI. The obvious trends in the expression of defence genes in these interactions, regardless of the viroid strain or host cultivar, led us to postulate that immune activation is most likely a result of the common mechanism of viroid pathogenesis. It has been suggested that viroids might elicit these immune responses due to their similarity with long noncoding RNAs (lncRNAs) [54,55]. LncRNAs are RNA molecules (>200 nt long), which are endogenous to host plants and may play a role in triggering plant defences by interfering with host RNA silencing components [56,57].

Other studies have suggested that viroids elicit immune responses through direct interactions of viroids with host proteins by an unknown mechanism (reviewed by [2,5]). HSVd, for example, has been shown to bind to cucumber phloem protein 2 (PP2) [58], while the avocado sunblotch viroid (ASBVd) was found to interact with two chloroplastic RNA-binding proteins [59]. The host proteins found to interact directly with viroids, thus far, seem to have distinctive functions in assisting in the spread or replication of the pathogens, and are specific to each interaction. Since no viroid-interacting proteins have yet been found to be conserved across all hosts, we surmise that it is unlikely that these direct interactions are responsible for the extensive activation of plant defence pathways across all viroid-infected plants.

## 3. Viroid Infection Interferes with Phytohormone Signalling Pathways

Phytohormones are a group of organic molecules found in all plants which act as chemical messengers to regulate various pathways within cells. These molecules were first noted for their critical roles in plant growth and development, but they have since become the focus of many plant–pathogen interaction studies due to their role in plant defence [18,60,61]. Different phytohormones have been revealed to be important in plant immunity against various pathogenic microorganisms [62,63], though their role in viroid–host interactions has only been highlighted in recent years. Several transcriptomic studies carried out in viroid-infected plants have noted significant changes to the expression of genes related to phytohormone pathways (Table 1).

### 3.1. Salicylic Acid

Salicylic acid (SA) is a key phytohormone in plant defence against biotrophic and hemibiotrophic pathogens, due to its role in the stimulation of programmed cell death (PCD) in response to pathogen attacks [64,65]. SA also functions in the regulation of various physiological processes, including seed germination, photosynthesis and growth, through its interaction with other phytohormones [66,67]. Upon viroid infection, several plant species showed changes in the expression of SA signalling-related genes, most of which were upregulated [9,10,24,29]. When the expression of specific genes involved in SA biogenesis was considered, however, their transcriptional activation seemed to be interaction-specific. Phenylalanine ammonia-lyase (*PAL*), for example, was upregulated in tomato roots in late PSTVd infection (49 days post-inoculation (dpi)) [24] and in sweet pepper cultivar Kurtovska kapia infected with PSTVd (mild symptoms) [35]. In contrast, *PAL* was repressed in PSTVd-infected hot pepper cultivar Djulunska shipka (severe symptoms) [35], and in a severe infection of tomato leaves with PSTVd at the stage of infection where symptoms first started to show (17 dpi) [28]. The unique expression profiles of *PAL* at distinct stages of symptom development in tomato, and in pepper cultivars with different symptom severities upon PSTVd-infection, suggest that the regulation of the SA pathway may be related to the appearance of symptoms in viroid disease.

### 3.2. Jasmonic Acid

Similar to SA signalling, the jasmonic acid (JA) pathway functions in the regulation of physiological processes in plants as well as in immunity. However, its interplay with the SA pathway means that components of JA signalling often have opposing expression profiles after pathogen infection [63,68,69]. Transcriptome data of plant hosts infected with viroids indicate that invasion by these noncoding pathogens affects the expression of genes involved in JA signalling [6,10,12,13,14,24,28,36,37]. An analysis of the expression profiles of specific genes in the JA pathway revealed that the regulation of this signalling pathway was, such as SA signalling, dependant on the viroid–host interaction being considered. This was unsurprising, given the antagonistic interaction between these pathways.

The lipoxygenase *(LOX)* gene family encodes enzymes which function early in the JA biosynthesis pathway [70]. Four different *LOX* genes showed increased expression in hop plants infected with HSVd and HLVd [37]. In contrast to this, all other viroid–host transcriptome studies that specifically analysed *LOX* expression found the gene to be downregulated [11,24,27,28]. It was noted by Takino and colleagues [11], however, that endogenous levels of JA were not altered in CSVd-infected chrysanthemum, despite the repression of *LOX*. This indicated that decreased *LOX* expression upon viroid infection may not necessarily influence the level of JA present in the infected plant.

*JAR1*, which encodes an enzyme responsible for the coupling of JA to amino acids such as isoleucine, had opposing expression patterns in viroid-infected hop depending on which plant tissues were sampled. This gene was downregulated in hop leaves infected with CBCVd or HLVd, though it was upregulated in CBCVd-infected hop flowers and in HLVd-infected hop cones [27]. *JAR1* also had intriguing expression profiles in tomato upon PSTVd infection. In the susceptible cultivar Rutgers, *JAR1* was upregulated in infected plants [18,28], while it was downregulated in the tolerant cultivar Moneymaker [6]. The duality of *JAR1* expression may be of importance when investigating mechanisms of tolerance and susceptibility of tomato to PSTVd. However, further analyses would be required to determine whether varied expressions of the JA biosynthesis genes coincide with an altered accumulation of endogenous JA levels in affected host plants.

### 3.3. Ethylene

Ethylene (ET) is a small signalling molecule linked to JA signalling with functions in plant growth and development, and responses to stress [71]. Unlike SA signalling, the ET pathway is known to act synergistically to the JA pathway [62,69]. The altered activation of the ET pathway, in general, was observed in viroid-infected hop, cucumber, sweet cherry, tomato and potato [6,9,13,14,24,27,28,29,33,37]. The expression analysis of specific ET pathway components, however, echoes the idiosyncratic expression patterns observed for SA and JA pathway components in viroid-infected hosts, where distinct transcriptional regulation has been observed depending on the host cultivar, tissue type and infection stage being considered.

*S-*adenosyl-L-methionine synthetase (*SAM1*), for example, which acts early on in ET biosynthesis, was shown to be repressed in PSTVd-infected tomato at 17 dpi, but transcriptionally activated at 49 dpi [24,28]. Two additional genes involved in ET biosynthesis, 1-aminocyclopropane-1-carboxylate (*ACC*) synthase (*ACS*) and ACC oxidase (*ACO*), were upregulated in tomato leaves infected with PSTVd [28] or CEVd [72], but downregulated in tomato roots after PSTVd infection [24]. *ACO* also had contrasting expression profiles in two different cultivars of pepper infected with PSTVd [35]. ET levels should be directly measured in viroid–host interactions to confirm whether the modified expression of ET biosynthesis genes results in altered concentrations of this hormone in planta. If ET is found to be differentially accumulated in specific viroid–host interactions with distinct symptoms, evidence would be provided to support the supposition that host ET levels are related to symptoms induced by viroid infection.

### 3.4. Auxin

Auxin is a key regulator of several physiological processes in plants, including growth, senescence, the formation of fruit and roots, leaf abscission, and stress responses [73,74]. Pathway analyses in viroid–host transcriptome studies suggest that auxin signalling and biosynthesis is altered in some plants upon viroid infection [9,10,11,12,13,14,24,28,33,36], though the activation or suppression of auxin pathway genes is not consistent across all interactions. The auxin-responsive *Aux/IAA* genes, for example, were revealed to be repressed in HSVd-infected sweet cherry, PSTVd-infected tomato and PLMVd-infected peach [8,28,36], but contrasting expression patterns were observed in tomato infected with the tomato planta macho viroid (TPMVd) or Mexican papita viroid (MPVd). RT-qPCR revealed that the up- or down-regulation of *IAA9*, *IAA3* and *ARF8b* (auxin responsive factor) was specific to the plant organs being studied [75]. Similarly, *ARF8* was upregulated in leaves, but not tubers, of PSTVd-infected potato [50].

It is plausible that variable expression patterns of auxin-related genes across viroid–host interactions may be linked to differences in symptoms observed for separate interactions, due to the role of auxins in physiological processes. One study suggested that the repression of auxin signalling genes may be related to the stunting symptom caused by CEVd infection of Etrog citron [12]. Other researchers correlated the downregulation of auxin pathway components with a reduced formation of lateral roots in PSTVd-infected tomato [24], further supporting a possible link between auxin signalling and symptoms of viroid disease.

### 3.5. Gibberellins

Gibberellins (GAs) are organic molecules which have long been known for their vital functions in plant growth and development by regulating various processes such as stem elongation, seed germination, flowering and fruit growth [60,76]. Only more recently has it been revealed that these chemical messengers also play a role in plant responses to various pathogens. It is yet to be established, though, whether GAs act by intensifying disease severity, or by aiding plant immune responses [77]. In terms of viroid–host interactions, transcriptome data support the former assertion, since the expression of GA biosynthesis genes in PSTVd-infected tomato is differentially regulated in different tomato tissues, at distinct stages of symptom development and in response to viroid variants of differing severity [24,28].

Contrasting expression patterns of GA pathway genes may indicate that changes observed here were associated with symptom development in viroid–host interactions. We hypothesise that the downregulation of the GA pathway in some viroid–host interactions is linked to the reduced plant growth that is a common symptom of Pospiviroid infection [11,78]. Reduced endogenous levels of GA linked to the suppression of GA biosynthesis genes in viroid–host interactions would provide strong evidence of this, similar to what has been demonstrated in CSVd-infected chrysanthemum [11]. Investigations into altered GA accumulation in other viroid-infected hosts, and, specifically, asymptomatic infections, would further support the possible link between GA signalling and viroid-induced symptoms.

### 3.6. Other Phytohormones

Additional phytohormone pathways that may be affected during viroid infection of plants are those related to biosynthesis and the signalling of brassinosteroids (BRs), abscisic acid (ABA) and cytokinins (CKs). The altered expression of genes involved in one or more of these phytohormone biosynthesis pathways, respectively, has been observed in viroid-infected tomato, sweet cherry, chrysanthemum and hop [6,12,14,24,28]. Similar to observations in other phytohormone pathways, the expression of components of BR-, ABA- and CK pathways appeared to be highly specific to the interaction being studied. Protein phosphatase 2C (*PP2C*), which negatively regulates ABA signalling, was downregulated in early infection (17 dpi) of tomato leaves with PSTVd [28], but upregulated in HSVd-infected sweet cherry fruit [36]. CK pathway genes were shown to have contrasting expression patterns in PSTVd-infected tomato depending on the type of variant used for infection, the host tissue being analysed and the different timepoints being investigated [24,28]. The lack of observable trends in phytohormone-related gene expression across different viroid–host interactions suggests that the activation or suppression of these pathways is linked to the specific symptoms observed for particular viroid diseases. Cellular levels of phytohormones should be compared between symptomatic and asymptomatic hosts following viroid infection. This may provide stronger evidence than transcriptome data for which phytohormones are linked to particular disease symptoms in specific hosts.

## 4. Pathways in Secondary Metabolism Are Affected by Viroid Infection

In addition to phytohormones, secondary metabolites (SMs) can also function as cellular messengers. SMs most notably mediate the interactions of plants with their environment and are not directly involved in normal growth processes [79]. SM production may be activated in response to abiotic stresses such as drought, light or changes in temperature, or it may be triggered in response to biotic stresses such as invasion by fungal, bacterial or viral pathogens [80,81]. Notable changes in the expression of genes related to SM pathways were observed in several viroid-infected hosts (Table 1), with most specific transcriptional changes being observed for the genes involved in the biosynthesis of flavonoids, terpenes and/or phenylpropanoids.

Flavonoids are phenolic compounds with diverse functions in plants, such as UV protection, the regulation of flower pigmentation, antioxidant activity and defence against invading microbes [82]. Genes involved in flavonoid biosynthesis were differentially expressed in Etrog citron infected with CEVd or citrus dwarfing viroid (CDVd) [12,83], in orchid infected with Dendrobium viroid (DVd) [25], in HSVd-infected sweet cherry and cucumber [10,36], in PSTVd-potato [34] and in hop infected with CBCVd or HLVd [13,14,84]. No common trends could be observed for the activation or suppression of this pathway across these studies, however.

Phenylpropanoids are known for their roles in plant defence—mostly due to their influence on the production of flavonoids, phytoalexins and lignin [85]. Lignin is a polymer that forms part of the secondary cell wall of plants, and its biosynthesis has been shown to be upregulated in response to pathogen attacks. Lignin monomers are produced using the phenylpropanoid pathway before lignin polymers are formed at the cell wall through a separate process [86,87,88]. Genes that encode enzymes in the phenylpropanoid pathway were mostly upregulated in CSVd infection of chrysanthemum [11] and in leaves and roots of PSTVd-infected tomato [24,28]. Authors of one study corroborated gene expression data by measuring the lignin content in infected tomato roots, confirming increased lignin accumulation in affected tissues [24]. Pathway analyses in other studies revealed that phenylpropanoid biosynthesis was downregulated in viroid-infected Etrog citron and cucumber [10,12], while changes to this pathway were variable in hop plants [13,14,89,90]. The fact that the expression of phenylpropanoid pathway genes is not similarly affected across viroid–host interactions suggests that the activation of this pathway may be linked to the phenotypic changes observed in infected plant hosts, rather than playing a role in host defence against viroids.

Terpenes compose a diverse group of organic compounds that are subdivided according to their chemical structures. In plants, these compounds may determine scent, attract insect pollinators, deter herbivores or play a role in defence against pathogens [91]. Several terpene biosynthesis-related genes showed modified expression in all hop-viroid interactions for which transcriptome data are available, as well as in viroid-infected potato and orchid [25,34]. However, no expression differences were observed in other viroid–host studies reviewed here. This suggests that modifications to the terpenoid pathway are specific to each particular viroid–host combination. Even in one particular host, terpene pathway genes showed divergent expression patterns, with terpene metabolism genes being mostly upregulated in hop infected with CBCVd and/or HLVd [13,14], and mostly downregulated in hop doubly infected with HSVd and HLVd [37]. The highly distinct alterations to the expression of terpene pathway genes in these interactions may be due to the different cultivars used in these studies, or they may be linked to the different symptoms observed in hop upon infection with different viroids. In one study, RT-qPCR was used to analyse the expression of key terpene biosynthesis genes in four different hop cultivars infected with HLVd [84]. Interestingly, the regulation of these genes seemed to be cultivar-specific, despite the symptomless nature of this interaction. In one of the cultivars, the authors also measured the cellular contents of certain terpenoids that were metabolised by the pathways they investigated. It was found that the accumulation of these terpenoids was altered in infected hop, but the changes to the terpenoid levels did not correlate to the changes in the expression of genes which influenced their metabolism [84].

## 5. Viroid Infection Alters Expression of Plant Cell Wall Components

In addition to the activation of immune signalling networks, and the use of chemical messengers/compounds to defend against pathogens, plants may also respond to microbial attack by physically altering components of host cells. The plant cell wall serves as a physical barrier to protect plants from microbes, and it has been observed to be modified in plants in response to pathogen invasion [92,93]. Besides its function in plant immunity, the cell wall also provides physical structure to host tissues, which means that the altered expression of cell wall-related genes upon a pathogen attack may be linked to the severity of disease symptoms rather than host defence—a fact which has been highlighted by a few researchers who analysed gene expression in viroid–host interactions [11,12,24]. Viroid-host transcriptome studies have shown that the expression of numerous cell wall-related genes was modified in several plant species (Table 1). These genes were mostly downregulated in the majority of these studies, which some authors linked to symptoms of malformation or stunted growth triggered by viroid infection [11,12,24].

Cellulose is a carbohydrate, composed of β-1,4-glucan, which confers structural strength to the plant cell wall [94]. In plants, cellulose synthesis is largely catalysed by cellulose synthases, while its hydrolysis is catalysed by endo-1,4-β-glucanases [95,96,97]. Transcriptomic studies have shown that PSTVd infection results in the modified expression of the genes encoding cellulose synthases and endo-1,4-β-glucanases in both the roots and leaves of tomato [24,28]. Another polysaccharide associated with cell wall-strengthening is callose, a β-1,3-glucan polymer that often accumulates to higher levels in response to pathogen infection [98]. A study by Rizza et al. [7] found that genes encoding callose synthases—which are primarily responsible for callose formation—were upregulated during the CEVd infection of Etrog citron. Authors supported gene expression changes with microscopy, which confirmed that viroid infection led to thickened cell walls and increased callose deposition [7]. In PSTVd-infected tomato, two callose synthases were shown to be targeted by vd-sRNAs, leading to the reduced accumulation of transcripts during infection [99]. Future studies should aim to investigate whether these genes are targeted in additional interactions to further elucidate the molecular interplay between viroids and their hosts.

The altered expression of genes encoding expansins was also noted during viroid infection [24,28]. Expansins are proteins without catalytic functions that have been shown to function in the loosening of the plant cell wall during various physiological processes [100,101]. The repression of expansin genes has been observed in the PSTVd infection of tomato and potato, as well as in the CSVd infection of chrysanthemum [11,24,28,50], but has not been noted specifically in other viroid–host interactions. The data for cell wall-related gene expression in viroid-infected hosts have revealed that modifications to the plant cell wall are largely dependent on the specific interaction being studied. The repression of cell wall-related genes in hosts that exhibit stunted growth or decreased root formation suggests that their altered expression in viroid-infected hosts is linked to symptoms of viroid infection, rather than plant defence against viroids [11,24].

## 6. Expression of Photosynthetic Components Is Altered by Viroid Infection

Photosynthesis is an essential process in the primary metabolism of plants, since the use of light energy to perform carbon fixation is the only way that plants can acquire the carbon they need to produce organic molecules within cells. Photosynthesis is, thus, linked to many other metabolic pathways in plants, as the carbohydrates produced during photosynthesis form the building blocks of many important cellular compounds such as amino acids, phytohormones and polymers that compose the plant cell wall [102,103]. It is, therefore, not surprising that photosynthesis has been differentially regulated when plants experience biotic stress [104].

Studies analysing transcriptomes of plants infected with viroids have shown significant changes in the expression of photosynthetic pathway components in some interactions (Table 1). Commonly altered photosynthesis-related genes in viroid–host interactions include genes encoding components of photosystems I and II, the thylakoid membrane and ATP synthases, as well as genes that code for proteins involved in chloroplast biogenesis, chlorophyll binding, carbon fixation, light reactions and oxidative phosphorylation. The expression of photosynthesis-related genes was downregulated in most studies, but, intriguingly, genes involved in photosynthesis were upregulated in the roots of tomato infected with PSTVd. Authors proposed that the transcriptional activation of photosynthetic genes in roots may be a reaction to decreased photosynthesis taking part in the plant, and might have a role in maintaining the balance between host immunity and plant growth in stressed tomato [24].

From a physiological perspective, it is understandable that photosynthesis would tend to be downregulated during viroid infection, since the activation of defence responses (as discussed earlier for these interactions) can be a costly exercise for plant hosts. One possibility is, thus, that photosynthesis is downregulated during infection to conserve energy needed for plant immune responses [14,102]. Another possibility is that the reduction in photosynthesis is linked to symptoms of viroid infection. The reduced accumulation of carbohydrates formed in photosynthesis would result in less cellular building blocks being available for growth, which could lead to stunting symptoms observed in some interactions. Additionally, the suppression of genes involved in chlorophyll metabolism could explain leaf chlorosis observed in viroid-infected hosts [10,13,14,29,35]. The changes observed in photosynthesis-related gene expression across different viroid–host interactions suggest that the inhibition of photosynthetic pathways may be of importance during plant responses to viroids in general, though it remains unclear whether this pathway is linked to the regulation of plant immunity or symptom expression in these interactions.

## 7. Transport Processes Affected in Viroid–Host Interactions

Transport proteins are involved in the movement of all chemical compounds throughout the plant—it is, therefore, reasonable to assume that genes encoding these proteins would be differentially expressed in plant–pathogen interactions, where multiple other processes are affected [105,106]. Plants infected with viroids have been shown to have altered expression of multiple genes encoding transporter proteins (Table 1), though this observation was not discussed as a major finding in most of these studies [8,9,11,13,14,28,29,35,36,37].

Transporters with modified transcription after viroid infection include ATP binding cassette (ABC) transporters, amino acid transporters, sugar transporters and ion transporters. ABC transporters are proteins that have important roles in the transport of phytohormones and secondary metabolites [107,108,109]. It is therefore plausible that their altered transcription in viroid-infected host plants may be linked to the shifting demand for these compounds in different plant tissues as the host responds to infection. Changes observed in the expression of genes encoding amino acid- and sugar transporters are also understandable, since plants infected with viroids may have varying needs for the chemical building blocks transported by these proteins [14]. Altered ion transport in viroid–host interactions may be linked to the activation of plant immunity upon viroid invasion, since ion transporters can influence the accumulation of inorganic ions inside host cells, which would impact ion exchange and the generation of ROS in affected hosts [9,110].

The fact that modified transport activity was common in almost all transcriptome studies reviewed here illustrates that changes to transport pathways seem to be a response shared by all viroid-infected hosts. Since none of these transcriptome studies analysed gene expression in transport pathways in detail, however, it remains unclear to what extent modified transporter activity plays a role in viroid–host interactions. Future studies into the magnitude of changes observed in transport pathways might help elucidate how viroid infection affects cellular transport in host tissues.

## 8. Viroid Infection Interferes with Processes in the Central Dogma

The phenotype of host plants is determined by the proteins present inside cells, of which the abundance is ultimately determined by the expression of genes that encode them. In eukaryotes, the post-transcriptional modification of mRNA transcripts, the translation of mRNA into proteins and the post-translational modification of proteins are all steps which may influence the accumulation of proteins. Additionally, the abundance of proteins is also affected by the rate at which they are degraded inside host cells at any given time. Transcriptome studies of viroid-infected hosts suggest that viroid infection may influence various steps in this process, from gene expression to protein degradation (Table 1).

### 8.1. Transcription Factors

The transcription of genes encoding TFs was shown to be affected in almost every global expression analysis of viroid-infected hosts (Table 1). This is unsurprising given the fact that the expression of genes is tightly regulated by TFs which bind to promoter elements upstream of the coding regions [111]. Thus, the altered expression of TF-encoding genes may account for the extensive changes to host transcriptomes upon viroid infection.

Several viroid–host studies noted changes in the expression of specific TF families. TF families showing altered expression levels following viroid infection most often include the bZIP family, bHLH family, MYB family, WRKY TFs, NAC TFs, C2H2 zinc fingers, ERFs and the C2C2-Dof family [12,13,14,24,28,29,35,36,84,89,90,112]. In general, these TF families have been shown to play a role in regulating the expression of genes involved in normal growth and developmental processes, plant defence, phytohormone signalling, secondary metabolism and plant response to abiotic stresses [113,114,115,116,117,118,119,120]. The differential expression of TFs could therefore be linked to the altered expression of genes involved in the various pathways and processes mentioned in this review. What remains unclear is how the modified expression of genes encoding TFs, which likely influences all other transcriptomic changes discussed here, is triggered by viroid infection. The expression of TFs may be altered due to the direct interaction of viroid RNA with specific host factors, or the adjusted DNA methylation status of TF genes due to the action of vd-sRNAs [2,4], though further investigations are needed to support these hypotheses.

### 8.2. RNA Processing

Eukaryotic pre-mRNAs undergo extensive modification before translation. One of the key processes in the post-transcriptional modification of RNA is RNA splicing, where noncoding regions of the RNA (introns) are removed, and coding regions (exons) are spliced together to form a mature RNA transcript. The process of alternative RNA splicing, where different combinations of exons are maintained in the unique versions of mature mRNA, can produce a number of different mRNA transcripts, and, therefore, different proteins.

One study on the transcriptome of PSTVd-infected tomato revealed that, in addition to the altered transcription of host genes, the mRNA of certain protein-coding genes was alternatively spliced upon viroid infection [9]. Authors noted that some genes related to plant stress responses, gene expression regulation and processes involved in biosynthesis and metabolism were altered in their splicing patterns in viroid-infected tomato compared to uninfected controls [9]. The changes in alternative splicing patterns observed present an opportunity for future research in other viroid–host interactions. The analysis of alternatively spliced transcripts in additional interactions would greatly increase our understanding of how viroid infection affects the host transcriptome on multiple levels prior to translation.

### 8.3. RNA Interference

Once mature mRNA has been produced, it is ready to be translated into a polypeptide chain. Through the process of RNA silencing, the abundance of mature mRNA transcripts may also be regulated. Briefly, this process involves the use of double-stranded RNA (dsRNA) or hairpin RNA (hpRNA) as a substrate for the enzyme Dicer or Dicer-like (DCL), which then cleaves the dsRNA or hpRNA into smaller RNA duplexes (sRNAs) 20–24 nucleotides long. These sRNAs may be classified as either small-interfering RNAs (siRNAs) or micro RNAs (miRNAs) depending on their size, function and interacting proteins. Once cleaved, sRNAs interact with Argonaute (AGO) proteins to form the RNA-induced silencing complex (RISC), which uses the sRNA to guide the complex to mRNA with a complementary sequence. AGO enzymes then cleave the targeted mRNA, effectively preventing its translation downstream [121,122,123,124].

Expression analyses in plants infected by viroids have shown marked changes in the expression of enzymes that are needed for RNA silencing. Authors noted the altered expression of *DCL* and *AGO* genes in HSVd-infected cucumber, PSTVd-infected tomato and CEVd-infected Etrog citron [10,12,29]. Additionally, a number of studies revealed that the expression of genes encoding certain RNA-dependent RNA polymerases (RDRs) was altered in viroid–host interactions. These enzymes are believed to amplify the effects of RNA silencing by increasing the abundance of the sRNAs used for targeting by RISC [125,126].

The effect of viroid infection on RNA silencing components remains unclear, as obvious trends are not easily observed in the available transcriptomic data. It is plausible that the altered expression of genes related to RNA silencing is linked to the mechanisms by which viroids cause disease (recently reviewed by [3,5]). Alternatively, it is possible that viroid infection influences gene silencing in a way that accounts for some of the downregulation that has been observed for many pathways discussed in this review. Investigations into vd-sRNAs produced by viroids during infection have found that, in some cases, the accumulation of specific vd-sRNAs could be linked to the downregulation of their target genes in host plants (summarised by [2]). Future research should more thoroughly investigate viroid interference with RNA silencing pathways in multiple viroid–host interactions. Where possible, the altered expression of host genes should also be linked to the presence of vd-sRNAs or plant sRNAs which target them. This would lead to an increased understanding of the significance of RNA interference triggered during viroid infection.

### 8.4. Translation

Analyses of affected pathways in transcriptomic studies showed that genes related to the process of translation were differentially expressed upon the viroid infection of sweet cherry and tomato [24,29,36]. Similarly, genes that encode ribosomal proteins, or that are related to ribosome biogenesis or metabolism, were noted to have altered expression in several different viroid–host interactions [6,13,14,24,33].

The majority of transcriptomic studies failed to note the modified expression of protein synthesis components as a key finding; however, when examined together, the combined transcriptomic data revealed that the manipulation of translation seemed to be common across various viroid–host combinations. To date, viroid interference with protein synthesis has not been specifically studied in any infected host, but altered levels of translation might mean that protein accumulation does not correlate to the level of gene expression observed in transcriptomic studies. Future investigations in this area should aim to support observations of differential gene expression with evidence of altered protein accumulation in affected host cells.

### 8.5. Protein Modification and Degradation

The abundance and activity of proteins may be affected by various post-translational processes. The differential expression of genes related to protein modification, protein phosphorylation and/or protein degradation has been observed in several hosts infected with viroids (Table 1). This suggests that protein activity (which is significantly influenced by the protein phosphorylation status) and protein accumulation (which is affected by the activation of protein degradation pathways) might be altered in plant hosts infected with viroids.

Early studies of viroid-infected hosts revealed the differential phosphorylation of a tomato protein upon PSTVd infection [127,128], and the modified phosphorylation of several proteins in tomato infected with CEVd [129]. However, more recent studies have not examined differences in the modification, phosphorylation and/or degradation of specific proteins in viroid–host interactions. A thorough investigation of altered protein abundance and activity upon viroid infection may provide evidence in support of the deductions determined based on transcriptomic data. If gene expression pathways are examined in their entirety, instead of considering cellular processes only in terms of their transcriptional activation, then the effect of viroid infection on plant phenotypes, and vice versa, could be better understood. Nevertheless, genome-wide transcriptome studies of viroid–host interactions remain valuable in that they present an informative overview of the more general possible effects of viroid invasion. These studies can then be used to indicate pathways of interest which future investigations can analyse in greater detail, ultimately leading to a deeper understanding of viroid–host interactions.

## 9. Viroid–Host Transcriptomics in Context and Avenues for Future Research

**Figure 1:**
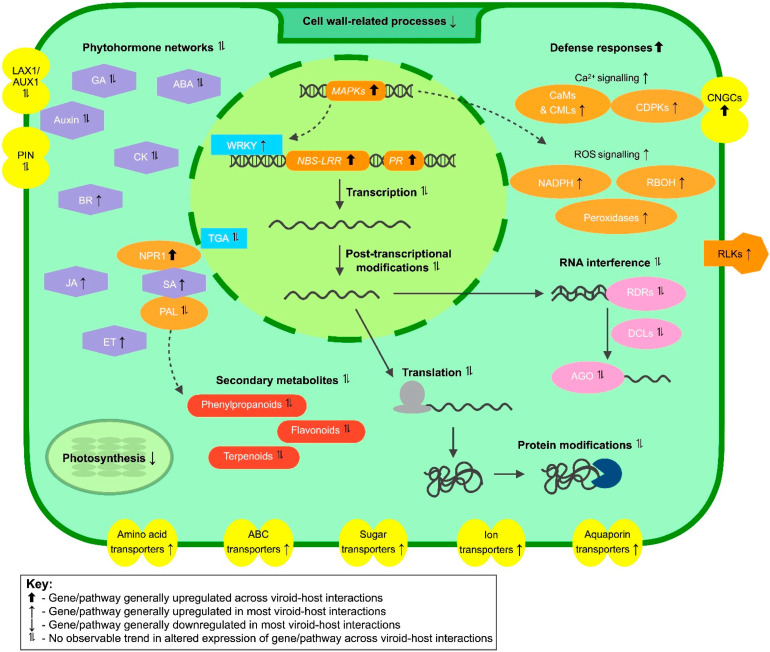
Signalling pathways and cellular components with altered gene expression after viroid infection. Transcriptome studies revealed that several functionally distinct gene groups were commonly affected in viroid–host interactions. Affected gene networks included those involved in plant immunity (*orange boxes*), phytohormone signalling (*purple boxes*), transport (*yellow boxes*), production of secondary metabolites (*red boxes*), cell wall modification and photosynthesis. Gene expression pathways were also affected, with components functioning in transcription (*light blue boxes*), post-transcriptional gene silencing (*pink boxes*), translation and protein modification commonly had altered expression profiles in viroid–host interactions. General trends in gene expression, based on available transcriptomic data, are indicated by the direction of arrows for each network/cellular component. Abbreviations: ABA—abscisic acid; ABC—ATP-binding cassette; AGO—Argonaute; AUX1/LAX1—auxin influx carrier protein; BR—brassinosteroid; CAM—calcium-binding calmodulin proteins; CDPK—calcium-dependent protein kinase; CK—cytokinin; CML—calmodulin-like protein; CNGC—cyclic nucleotide-gated channels; DCL—Dicer-like protein; ET—ethylene; GA—gibberellic acid; JA—jasmonic acid; MAPK—mitogen-activated protein kinase; NBS-LRR—nucleotide binding site leucine-rich repeat protein; NPR1—nonexpressor of pathogenesis-related 1; PAL—phenylalanine ammonia-lyase; PIN—auxin efflux carrier protein; PR—pathogenesis-related genes; RBOH—respiratory burst oxidase homolog; RDR—RNA-dependent RNA polymerase; RLK—receptor-like kinase; ROS—reaction oxygen species; SA—salicylic acid.

The molecular mechanisms of viroid pathogenesis have been the subject of scientific investigations for over 20 years, yet much remains unknown. Several recent studies have analysed host gene expression in response to viroid infection in the hopes that this would further our understanding of these interactions. When the results of viroid–host transcriptomic studies were taken together, several cellular components and signalling networks were observed to be commonly influenced by viroid infection (Figure 1).

The most distinctive trends in gene expression were observed for plant defence pathways, which were upregulated across all viroid–host interactions. Hormone signalling pathways appeared to be affected in all viroid-infected hosts (Table 1), though the modified transcription of the phytohormone-related genes is highly specific to each interaction being studied. With the exception of plant immune pathways, transcription level alterations in all gene networks were found to differ between separate host cultivars, different host tissues being examined, specific time points at which sampling occurred and the severity of the variant/s used in each study.

Currently, the transcriptomic response of tomato to PSTVd is the only interaction studied in depth, with all variables taken into consideration. Only one or two transcriptomic studies have been carried out for each of the other viroid–host interactions reviewed here (Table 1), while host responses to some viroids have not been studied at all. Investigating more viroid-infected hosts with the use of transcriptomic data and taking multiple variables into account would allow stronger conclusions to be drawn regarding commonalities and differences between these interactions. The availability of transcriptome data for additional interactions would also allow distinctions to be determined in terms of whether the altered gene expression for specific pathways is due to the host responding to the infection, or if it is linked to specific symptoms observed for each interaction. Upon a review of the data currently available, it appears that transcript level changes of host genes not relating to plant defence are likely correlated to disease development during viroid infection.

Host gene expression is extensively modified during viroid infection, but it remains unclear how this expression is triggered by what Flores et al. [3] termed “the initial molecular lesion”. In their review, authors put forward the idea that viroid pathogenesis should be studied in terms of the distinct symptoms caused by different viroid families, and that the RNA silencing of specific target mRNAs could only be directly linked to symptoms caused by members of *Avsunviroidae* [3]. It is conceivable, however, that gene expression changes in Pospiviroid infection may be due to the action of *trans*-acting siRNAs, which would trigger signalling cascades in host cells [2,130]. Another hypothesis that has recently been put forward by Gómez et al. [131] states that host transcriptional activity might be altered due to changes in the plant epigenome induced by Pospiviroids.

One theory that has very recently been proposed is that viroids, which have always been considered noncoding entities, may have the capacity to encode peptides after all [132]. This has been supported by the discovery of open reading frames (ORFs) within some viroid genomes, and the association of HSVd and eggplant latent viroid (ELVd) with translational machinery in planta [132]. PSTVd has also been revealed to associate with ribosomes in tomato and *Nicotiana benthamiana* [133]. Despite this association, peptides produced by PSTVd were not found in those hosts. Future studies would, therefore, need to investigate whether peptides are produced by other viroids in additional hosts, and whether these might be involved in activating downstream changes in host gene expression. Alternatively, viroid RNA itself may act as a PAMP to trigger defence responses independent of RNA silencing pathways [4,44], which would then influence downstream gene expression pathways.

While transcriptomic studies are incredibly valuable in elucidating the host response to viroids, gene expression data alone are not enough to draw solid conclusions regarding the effects of viroid infection. The host phenotype is influenced by the abundance of specific proteins, but the transcription of a particular gene might not necessarily correlate with the accumulation or activity of the encoded protein. Conclusions drawn from transcriptome data should, thus, be supported by evidence of altered protein functioning. Milanović et al. [50] used RT-qPCR to analyse gene expression in PSTVd-infected potato, but also measured endogenous levels of specific hormones, antioxidants and chlorophyll content to better understand this interaction. A recent study in hop compared the secondary metabolite contents in healthy and HLVd-infected plants before using RT-qPCR to analyse the expression of genes involved in the biosynthesis and regulation of those metabolites [84]. These studies demonstrate the value of more detailed investigations into specific aspects of host response. Future studies should use multiomics approaches to shed light on viroid–host interactions. Investigating the metabolome and proteome of hosts for which transcriptomic data are already available would build on the knowledge gained in these interactions to date, and strengthen conclusions that have been drawn regarding the correlation of gene expression with specific disease symptoms.
